# Roles of lncRNAs in pancreatic beta cell identity and diabetes susceptibility

**DOI:** 10.3389/fgene.2014.00193

**Published:** 2014-07-01

**Authors:** Timothy J. Pullen, Guy A. Rutter

**Affiliations:** Section of Cell Biology, Department of Medicine, Imperial Centre for Translational and Experimental Medicine, Imperial College LondonLondon, UK

**Keywords:** type 2 diabetes, genome-wide association studies, lncRNAs, beta cell, islets of Langerhans, cell identity

## Abstract

Type 2 diabetes usually ensues from the inability of pancreatic beta cells to compensate for incipient insulin resistance. The loss of beta cell mass, function, and potentially beta cell identity contribute to this dysfunction to extents which are debated. In recent years, long non-coding RNAs (lncRNAs) have emerged as potentially providing a novel level of gene regulation implicating critical cellular processes such as pluripotency and differentiation. With over 1000 lncRNAs now identified in beta cells, there is growing evidence for their involvement in the above processes in these cells. While functional evidence on individual islet lncRNAs is still scarce, we discuss how lncRNAs could contribute to type 2 diabetes susceptibility, particularly at loci identified through genome-wide association studies as affecting disease risk.

## INTRODUCTION

Diabetes is a major and growing health problem affecting 347 m people worldwide (Danaei et al., 2011). Type 2 diabetes (T2D) accounts for 90% of affected individuals, and is a complex, progressive disease affected by a range of genetic and environmental risk factors. While insulin resistance plays a part in disease progression, pancreatic beta cell failure lies at the heart of T2D (Kahn, 2003).

Beta cells are the body’s sole source of circulating insulin, and as such are critical for maintaining blood glucose within healthy limits. The beta cell’s tightly regulated secretion of insulin in response to glucose is dependent on a highly specialized metabolic sensing system, underpinned by a specific pattern of gene expression. This includes specific genes required for glucose-sensing (e.g., *Gck* and *Glut2*), insulin production and processing (e.g., insulin, prohormone convertase 1/3 [PC1/3]) and regulated secretion. Equally important is the specific repression of genes which interfere with proper regulation of insulin secretion (e.g., *Slc16a1* and *Ldha*) (Zhao et al., 2001; Pullen et al., 2012). Indeed, we (Pullen et al., 2010) and others (Thorrez et al., 2011) have recently identified ∼40 “forbidden” or “disallowed” genes specifically repressed in islets despite widespread expression across other tissues. While a unique pattern of transcription factors (e.g., Pdx1, Pax6, MafA, Nkx2.2) likely underlie the sustained expression of beta cell selective genes, epigenetic mechanisms are vital for the mitotically stable maintenance of cellular identity and probably the suppression of forbidden genes.

The tipping point from pre-diabetes to overt T2D is usually reached when beta cells can no longer compensate sufficiently for incipient insulin resistance. The nature of the progressive loss of functional beta cell mass has been the source of some debate (Weir and Bonner-Weir, 2013), with both decreases in overall beta cell mass (Butler et al., 2003) and impaired glucose-stimulated insulin secretion (GSIS) from the remaining islets being reported in T2D subjects (Del Guerra et al., 2005). Indeed, recent work has suggested that decreases in beta cell mass are modest (Rahier et al., 2008) and may even have been over-estimated (Marselli et al., 2014).

There has also been growing interest in a third option: that a progressive loss of beta cell identity underlies the development of T2D. Results from two rat models (Tokuyama et al., 1995; Jonas, 1999), and the *db/db* mouse model (Kjorholt et al., 2005) of T2D produce similar results showing a loss of differentiated beta cells accompanied by similar changes in gene expression: decreased expression of GSIS genes (e.g., *Gck*); increased expression of normally repressed genes (e.g., *Ldha* and *Hk1*); and decreases in islet transcription factors (e.g., *Isl1*, *Neurod1*, *Pdx1*, and *Pax6*) (Tokuyama et al., 1995; Jonas, 1999). The importance of these transcription factors in maintaining beta cell identity is underscored by the transformation of beta to alpha-like cells following *Pdx1* deletion (Gao et al., 2014). In *FoxO1* knockout mouse islets, beta cells dedifferentiate a step further, acquiring expression of immature endocrine cell markers (*Ngn3*, *Oct4*, *Nanog*, and *Mycl*) (Talchai et al., 2012). However, the relevance of this strain to human diabetic islets is as yet unestablished.

While the relative importance of these three factors (impaired beta cell mass, function and identity) in T2D is not fully understood, mechanisms which increase beta cell function, proliferation and identity are all sought to tackle the disease. Furthermore, increased understanding of the molecular defects underlying T2D will enable novel therapies to address the underlying causes of the disease, rather than just treat the symptoms.

A novel layer of gene regulation, acting partly through epigenetic mechanisms, was uncovered with the discovery that thousands of long non-coding RNAs (lncRNAs) are expressed from the genome (Guttman et al., 2009). lncRNAs are a diverse group of transcripts defined by a negative property: the lack of protein-coding potential. They are differentiated from short ncRNAs such as miRNAs (which also play an important role: see Poy et al., 2004; Guay et al., 2011; Pullen et al., 2011) by a minimum length threshold of 200 nt. lncRNAs are often capped, spliced and polyadenylated like mRNAs although both unspliced and non-polyadenylated variants are also common. As a class, lncRNAs are enriched in the nucleus relative to protein-coding transcripts, although there remains a population of cytoplasmic-enriched lncRNAs, and non-nuclear functions have been demonstrated for a number of lncRNAs (Derrien et al., 2012). Indeed lncRNAs have been demonstrated to regulate gene expression through a number of mechanisms at the transcriptional, post-transcriptional and translational level.

The expression patterns of lncRNAs are significantly more cell-type specific than protein-coding genes (Guttman et al., 2009; Djebali et al., 2012), making them well placed to play highly cell-type specific roles. However, the fact that lncRNAs are less evolutionarily conserved at the primary sequence level than protein-coding genes and the fact the numerous lncRNAs appear to be species-specific raise questions over their functions. lncRNAs may depend more on secondary structure for their function, and thus be more tolerant of mutations than protein-coding genes. Indeed, lncRNA exons show evidence of low but clear sequence conservation compared to other intergenic regions (Guttman et al., 2009). The advent of next generation sequencing technologies has led to an explosion of novel lncRNA discovery in diverse tissues, including pancreatic islets and beta cells. The discovery of lncRNAs with the potential to regulate gene expression and cellular identity in T2D-relevant tissues offers the opportunity for greater understanding of disease etiology and novel targets for treatment.

## MECHANISMS OF lncRNA ACTION

miRNAs are a major class of short non-coding RNA which primarily act via a single, well-characterized mechanism to downregulate gene expression through mRNA degradation and translational inhibition. In contrast, lncRNAs have been shown to regulate gene expression through a bewildering array of mechanisms (reviewed extensively in Wang and Chang, 2011; Kung et al., 2013). On average, lncRNAs are less abundant than protein-coding transcripts, although some (e.g., *H19*) are expressed at comparable levels (Djebali et al., 2012). lncRNAs are a heterogeneous group of transcripts, and greater functional characterization will hopefully allow clearer classification into distinct groups acting through distinct mechanisms.

One of the first identified modes of action of lncRNAs was via alterations in chromatin modifications through recruiting histone modifying complexes to particular genomic loci (Rinn et al., 2007). This plays a major part in epigenetic silencing during X-inactivation (through the lncRNA *Xist* (Zhao et al., 2008; Pontier and Gribnau, 2011)) and at imprinted loci (e.g., *Air* at the *Slc22a3* locus (Nagano et al., 2008)). Indeed, this appears to be a widespread function of lncRNAs, with around 20% of intergenic lncRNAs identified in one study interacting with the chromatin-modifying complex Polycomb Repressive Complex 2 (PRC2) (Khalil et al., 2009). In addition to affecting histone modifications, lncRNAs can also act on the other major branch of epigenetic regulation, DNA methylation, through interaction with DNA methylation machinery (Mohammad et al., 2010; Di Ruscio et al., 2013). lncRNAs can further regulate gene expression by mediating DNA looping (Lai et al., 2013). This affects the three-dimensional conformation of chromosomes which regulates gene expression by bringing distal enhancers physically adjacent to gene promoters.

lncRNAs also act through a number of mechanisms to regulate gene expression at the post-transcriptional level. Natural antisense transcripts (NATs) are lncRNAs transcribed in the opposite orientation to mRNAs with overlapping exons. They have the potential for direct interaction with mRNAs through complimentary base-pairing, which can increase mRNA stability (e.g., *BASE1-AS* and *BASE1*) (Faghihi et al., 2008). In contrast, lncRNAs can also lead to mRNA degradation through the recruitment of Staufen1 (STAU1) (Gong and Maquat, 2011). lncRNAs can also influence mRNAs indirectly by acting as “sponges” for miRNAs, preventing them from downregulating gene expression (Kallen et al., 2013). It is interesting to note that this last example refers to the highly expressed *H19* lncRNA, and these post-transcriptional mechanisms presumably require lncRNAs in comparable abundance to their mRNA or miRNA targets. Given the lower expression of most lncRNAs relative to mRNAs these mechanisms requiring abundant lncRNAs are perhaps the exception rather than the norm.

## lncRNAs AND BETA CELL IDENTITY

As the expression of many lncRNAs is highly cell-type specific, early catalogs of lncRNAs mainly from other cell lines provided little information relevant to beta cells. However, a number of studies have since identified lncRNAs genome-wide in human and mouse, islets and beta cells.

Morán et al. (2012) used combination of ChIP-Seq (to identify sites of active transcription), and RNA-Seq (to directly detect transcripts) to produce a comprehensive catalog of >1100 lncRNAs expressed in human islets. In agreement with studies in other tissues, these lncRNAs proved to be significantly more cell-type specific than protein-coding genes. Indeed, 55% of intergenic lncRNAs and 40% of antisense lncRNAs identified in this study were specific to islets. Importantly, Morán et al. (2012) identified several lncRNAs that were dysregulated in islets from T2D subjects.

One challenge to the theory of widespread functions for lncRNAs is the low level of sequence conservation between species. To address this, orthologous mouse genomic regions were detected for 70% of human lncRNA loci, and RNA-Seq revealed that 47% of these were actively transcribed in mouse islets (Morán et al., 2012). Indeed this may be an underestimate as several lncRNAs were detected by qPCR despite falling below the threshold for RNA-Seq detection. An independent transcriptomic study of mouse islets identified a similar number (>1000) of intergenic lncRNAs (Ku et al., 2012). Further dissection of islet cell-types was performed by Bramswig et al. (2013), with the identification of five alpha cell-specific and twelve beta cell-specific lncRNAs. The scarcity of lncRNAs identified in this study may be due to highly stringent removal of any transcripts overlapping repeat regions.

Previous studies have identified both lncRNAs that regulate the maintenance of pluripotency, and lncRNAs required for neuronal differentiation (Ng et al., 2012). Indeed, the regulation of differentiation and cell identity appears to be a major function of lncRNAs. Supporting this role for islet lncRNAs, Morán et al. (2012) identified dynamic regulation of lncRNAs during *in vitro* differentiation of human embryonic stem cells towards pancreatic endocrine cells. As such protocols have thus far failed to produce fully functional beta cells without an *in vivo* maturation stage in mice, perhaps the most tantalizing discovery was six lncRNAs whose expression was only activated during this last stage. Understanding regulators of this critical maturation stage, and the ability to recapitulate it without passage through animals would be essential for any therapeutic generation of beta cells from human stem cells. However, islet lncRNAs are not restricted to developmental roles, as depletion of a beta cell-specific lncRNA was also shown to influence gene expression in mature beta cells. Depletion of the lncRNA *HI-LNC25* decreased expression of the transcription factor and monogenic diabetes gene, *GLIS3* (**Table 1**) (Morán et al., 2012). The location of *HI-LNC25* and *GLIS3* on separate chromosomes (20 and 9, respectively) indicates that the lncRNA regulates *GLIS3* in *trans*, although whether this is through a direct interaction with this locus has not been explored.

**Table 1:** The main lncRNAs discussed in this paper along with their tissue and species specificity.

lncRNA	Islet-specific expression	Mouse ortholog identified	Reference
*HI-LNC25*	Yes	No	Morán et al. (2012)
*KCNQ1OT1*	No	Yes	Travers et al. (2013)
*CDKN2B-AS1 (ANRIL)*	No	No – although a lncRNA has been reported at the orthologous locus	Cunnington et al. (2010), Pasmant et al. (2011), Visel et al. (2010)

## lncRNAs IMPLICATED IN T2D SUSCEPTIBILITY

Within the last decade, genome-wide association studies (GWAS) have been a major focus of work to identify the genetic variants underlying susceptibility to T2D and related metabolic traits. Through identifying single nucleotide polymorphisms (SNPs) which correlate with diabetic phenotypes, investigators aim to single out genes which influence disease susceptibility. While much of the interpretation of GWAS hits has focussed on protein-coding genes, there are good reasons to indicate that lncRNAs are responsible for the effects of some of these SNPs.

It is striking how few SNPs identified through GWAS for T2D, indeed for most diseases, result in changes to protein sequences. *SLC30A8* is one of the few examples from early studies where a SNP (rs13266634) resulted in an amino acid substitution (R325W) affecting the zinc transporter located on insulin granules (Sladek et al., 2007). Interestingly, expression of *SLC30A8* is largely restricted to pancreatic islets, so the effects of any mutations are expected to be limited to the endocrine pancreas. In contrast, genes at most other GWAS loci are more widely expressed. Missense mutations at these widely expressed loci are far more likely to cause defects in multiple tissues and possibly embryonic lethality, which could account for absence from GWAS specific for T2D and related traits. In a more recent large-scale meta-analysis which aimed to finely map the causal SNPs, only two of the 65 T2D susceptibility loci examined had a lead SNP resulting in a missense mutation (*PPARG* [rs1801282] and *KCNJ11* [rs5215]) (Morris et al., 2012). In both cases, rare severe mutations have previously been identified which cause monogenic forms of diabetes (Barroso et al., 1999; Gloyn et al., 2004). Most SNPs instead map to intronic or intergenic regions and thus likely act through altering gene expression and/or splicing.

While the effects of changes to protein sequences are relatively straightforward to investigate, it is often unclear which genes are affected by SNPs in non-coding regions. Whereas proximal promoters are directly adjacent to the genes they regulate, enhancer elements can influence the expression of genes hundreds of kilobases away, and can be interspersed between and even within other genes (Ilnytska et al., 2009). Interestingly, SNPs associated with T2D and fasting glycaemia are enriched in these distal regulatory regions (Pasquali et al., 2014). The gene affected by SNPs falling outside proximal promoters may not be clear, although the closest gene is often used as a starting point for further investigation. With the identification and annotation of increasing numbers of lncRNAs it has become apparent that a number of the GWAS hits fall close to, or within lncRNAs.

Mapping SNPs at these 65 loci to current Ensembl annotations shows five lead SNPs are within the exons or introns of lncRNAs (**Table 2**). Two of these loci (*KCNQ1* and *CDKN2A/CDKN2B*) contain well characterized lncRNAs for which studies have started to reveal the mechanisms through which they function. At the *PROX1* and *PSMD6* loci, in both cases the lead SNP (rs2075423 and rs12497268) falls within the exon of an annotated antisense orientated lncRNA. At the *CCND2* locus, the lead SNP (rs11063069) is approximately 9 kb upstream of the protein-coding gene, yet within the intron of two antisense lncRNAs (*CCND2-AS1* and *CCND2-AS2*). Finally, as further islet-expressed lncRNAs are identified more of these SNPs are found to fall closer to lncRNAs than protein-coding genes (e.g., *WFS1* locus (Morán et al., 2012)).

**Table 2:** Type 2 diabetes susceptibility loci where a lead SNP falls within a lncRNA gene.

SNP	Locus	Result of combined meta analysis (Morris et al., 2012)	Annotation closest to SNP	Gene type	lncRNA Name
rs2075423	*PROX1*	Lead SNP	Non-coding exon	Antisense lncRNA	*PROX1-AS1*
rs12497268	*PSMD6*	Lead SNP	Non-coding exon*	Antisense lncRNA	*PRICKLE2-AS1*
rs10811661	*CDKN2A/B*	Lead SNP	13.5 kb downstream of lncRNA gene	Antisense lncRNA	*CDKN2B-AS1 (ANRIL)*
rs944801		Lead SNP for putative secondary signal	Intron	Antisense lncRNA	*CDKN2B-AS1 (ANRIL)*
rs163184	*KCNQ1*	Lead SNP	Intron	Protein-coding	*KCNQ1*
rs231361		Lead SNP for putative secondary signal	Non-coding exon*	Antisense lncRNA	*KCNQ1-OT1*
rs11063069	*CCND2*	Lead SNP	Intron	Antisense lncRNA	*CCND2-AS1 / CCND2-AS2*

SNPs reported in a meta analysis of GWA studies (Morris et al., 2012) overlapping lncRNA annotations in Ensembl (assembly GRCh38). *SNP also falls within an intron of the protein-coding gene.

### KCNQ1

The *KCNQ1* locus contains a number of genes with the potential to affect beta cell function and proliferation. Within this locus *KCNQ1*, *KCNQ1OT1*, *CDKN1C*, *PHLDA2*, *SLC22A18*, and *SLC22A18AS* are all expressed in both fetal pancreas and adult islets (Travers et al., 2013). *KCNQ1* encodes a voltage-gated potassium channel subunit which is expressed in pancreatic beta cells although its effect on the regulation of insulin secretion is somewhat unclear. siRNA knockdown of *KCNQ1* in human islets enhanced depolarization-induced exocytosis (Rosengren et al., 2012), whereas pharmacological inhibition in INS-1 cells did not affect basal, tolbutamide or GSIS (Ullrich et al., 2005). *CDKN1C* encodes the p57*^KIP2^* cyclin-dependent kinase inhibitor which is a negative regulator of cell proliferation. It is expressed in islets, and loss of p57*^KIP2^* expression is associated with increased beta cell proliferation in focal congenital hyperinsulinism (Kassem et al., 2001; Henquin et al., 2011). *PHLDA2* also exerts a negative effect on cell proliferation, with increased expression in the placenta being associated with intrauterine growth retardation though uteroplacental insufficiency (McMinn et al., 2006). In contrast, decreased expression of *PHLDA2* was detected in neuroendocrine tumors relative to normal islet controls, as a downstream effect of losing the tumor suppressor gene *MEN1* resulting in Multiple Endocrine Neoplasia type 1 (Dilley et al., 2005).

The lead SNP at this locus is within the intron of the *KCNQ1* protein-coding gene, yet the lead SNP for a putative secondary association signal is in the exon of an antisense lncRNA (*KCNQ1OT1*) (Morris et al., 2012). *KCNQ1OT1* is a 91 kb long transcript encoded by RNA polymerase II and localized exclusively in the nucleus (Pandey et al., 2008). In early development, *KCNQ1OT1* is expressed in a monoallelic fashion from the paternal allele and has been linked to silencing of nearby genes on the same chromosome, resulting in these genes being expressed exclusively from the maternal allele. This is mediated through the interaction of *KCNQ1OT1* with PRC2 components and G9a histone methyltransferase resulting in enrichment of repressive histone modifications H3K27me3 and H3K9me3 at this locus (Pandey et al., 2008). However, this pattern is complicated by the lineage-specific loss of imprinting at some genes. Specifically, *KCNQ1* and *KCNQ1OT1* showed developmental loss of imprinting with biallelic expression in adult islets (Travers et al., 2013). Interestingly, in the developing mouse heart, *Kncq1ot1* is required for paternal silencing of *Cdkn1c* and* Slc22a18*, but not *Kcnq1* indicating that *Kcnq1ot1* may be more directly involved in the regulation of these two genes (Korostowski et al., 2012).

One proposed model to explain the effect of SNPs at this locus is that risk alleles reduce *KCNQ1OT1* expression, thereby decreasing repressive histone modifications, increasing *CDKN1C* expression and thereby impairing islet proliferation or development (Travers et al., 2013). In contrast to this model, *KCNQ1OT1* was reported to be upregulated in T2D islets (Morán et al., 2012). However, this apparent contradiction may be explained by risk SNPs having an effect on imprinting early in development, whereas the *KCNQ1OT1* upregulation in T2D adult islets may be part of the compensatory proliferative response of islets to hyperglycaemia.

### ANRIL

*CDKN2A*/*CDKN2B* is another T2D GWAS locus containing a well-characterized lncRNA. This locus encodes three tumor-suppressors: p16*^INK4A^*, p14*^ARF^*, and p15*^INK4B^*. The expression of these genes is regulated by polycomb-mediated silencing through H3K27me3. Of particular relevance to diabetes, p16*^INK4A^* up-regulation has been shown to be responsible age-dependent decline in beta cell proliferative capacity (Krishnamurthy et al., 2006). The histone methyltransferase and PRC2 component, Ezh2, represses p16^INK4A^ expression in young beta cells, permitting proliferation. However, declining Ezh2 levels in aging beta cells lead to derepression of p16^INK4A^ expression, and consequent inhibition of proliferation (Chen et al., 2009).

The lead SNP (rs10811661) at this locus is downstream of, and closest to the lncRNA *ANRIL* (*CDKN2B-AS1*). The lead SNP for a putative secondary association signal (rs944801) is located within an intron of *ANRIL* (Morris et al., 2012). This region (chromosome 9p21) has been strongly associated with susceptibility to a number of diseases. SNPs associated with coronary disease, stroke, melanoma and glioma are all located close to *ANRIL* and correlated with *ANRIL* expression (Cunnington et al., 2010; Pasmant et al., 2011). Although no direct mouse ortholog of *ANRIL* has been reported, a lncRNA of unknown function has been detected at the orthologous region of the mouse genome. Deletion of part of the “9p21” orthologous region in mice (including a section of this lncRNA) increased mortality and affected expression of neighboring genes (Visel et al., 2010), indicating that gene regulatory sequences and possibly this lncRNA perform roles conserved between human and mouse.

*ANRIL* interacts with both PRC1 (CBX7) and PRC2 (SUZ12) components to affect the epigenetic regulation of gene expression at this locus (Yap et al., 2010; Kotake et al., 2011). Competitive inhibition of *ANRIL* was reported to increase *p16^INK4A^* expression resulting in decreased proliferation in fibroblasts (Yap et al., 2010). A separate depletion of *ANRIL* in lung fibroblasts also reported decreased proliferation, although in this case primarily through upregulation of *p15^INK4B^*. The direction of this effect fits with reports of T2D SNPs being associated with decreased *ANRIL* expression, although not in all populations (Cunnington et al., 2010). It therefore appears that *ANRIL* recruits both PRC1 and PRC2 to this locus in a coordinated manner to repress these proliferation inhibitors. Disruption of *ANRIL* expression or function by SNPs could impair silencing at this locus decreasing the proliferative capacity of beta cell.

One should caution that *ANRIL* may not be limited to cis-acting effects, as overexpression of *ANRIL* in HeLa cells has also been reported to produce genome-wide effects on gene expression (Sato et al., 2010). An interesting direction for future research may thus be to determine how regulation of this locus by *ANRIL* integrates with the PDGF pathway responsible for age-dependent decline in beta cell proliferative capacity (Chen et al., 2011). Whereas ectopic expression of *Ezh2* was sufficient to increase beta cell proliferation in young mice, it was unable to repress *p16^INK4A^* in older mice without combined inhibition of trithorax group proteins (Zhou et al., 2013). It would be of particular interest to discover whether manipulation of *ANRIL* in adult beta cells can reverse this block on proliferation, or whether its effects are primarily in developing beta cells.

It is interesting to note that *ANRIL* and *KCNQ1OT1* are both expressed in numerous tissues, yet certain SNPS within them are associated specifically with T2D susceptibility. It may be that beta cell replication during compensation to insulin resistance is particularly sensitive to *ANRIL* function meaning that these cells are most severely affected by *ANRIL* variants. Alternatively, *ANRIL* may be involved in beta cell-specific interactions which are uniquely affected by T2D SNPs. While the former proposition appears more likely, functional studies from one cell-type may not be directly applicable to others. The lack of a clear mouse ortholog of *ANRIL* has also prevented the use of mouse transgenics for investigating its function. Further investigation into the relationship between lncRNAs expressed in human and mouse at this locus would provide valuable insight into whether mouse experiments could be used to study *ANRIL* function.

## lncRNAs AS THERAPEUTIC TARGETS

The discovery of lncRNAs implicated in susceptibility to, and the progression of, T2D raises the question of whether lncRNAs can be therapeutically manipulated to ameliorate this disease. lncRNAs offer a number of advantages as therapeutic targets. Firstly, the highly cell-type specific expression pattern of many lncRNAs is likely because they are involved in the cell-type specific regulation of genes which themselves are more widely expressed. Manipulating lncRNAs could therefore allow the effects to be specifically targeted to a single cell-type, with few side effects.

Furthermore, lncRNAs are involved in the epigenetic regulation of fundamental cellular processes such as pluripotency and cell identity. Much of the protein machinery regulating the epigenetic landscape is widely expressed, making targeting any intervention difficult. By acting as scaffolds to bring together particular combinations of chromatin-modifying complexes, transcription factors, etc. and target them to specific genomic loci, lncRNAs can provide specificity to this system. As such, lncRNAs may also provide a specific target to influence the epigenetic landscape underlying beta cell identity, and the potential to reinforce beta cell identity when this is challenged by T2D.

In contrast to lncRNAs, the more clearly defined mechanisms of miRNA processing and function have allowed greater progress to be made towards therapeutic targeting of these short non-coding RNAs. The most successful route has been to inhibit miRNAs *in vivo* by administrating modified oligonucleotides systemically (Montgomery and van Rooij, 2011). Although the short length of miRNAs makes them easy to target using short complementary oligonucleotides, a similar approach termed “antagoNATs” uses short antisense oligonucleotides to target lncRNAs. Brain-derived neurotrophic factor* (BDNF-AS)* is a brain expressed NAT which inhibits the *BDNF* gene. Continuous *in vivo* delivery to mouse brain via an osmotic minipump stably increased BDNF expression in a highly locus-specific effect, with a corresponding increase in neurite outgrowth and differentiation (Modarresi et al., 2012). While this form of delivery may be of limited use in clinics, advances in delivery of other modified oligonucleotides, such as siRNAs, will likely increase the feasibility of using this approach more widely (Gavrilov and Saltzman, 2012). Whether similar approaches could be used to target diabetic beta cells relies on the identification and characterization of islet-specific lncRNAs with negative impacts on beta cell identity, function or proliferation.

Therapeutic increases in miRNA function have been possible using chemically synthesized miRNA mimics. While the mimics themselves are highly effective, targeting their delivery to a particular cell type is not. The use of adeno-associated viral (AAV) vectors offers the possibility to target miRNA expression to particular tissues through both viral tropism and the use of tissue-specific promoters (Montgomery and van Rooij, 2011). Synthetic lncRNA mimics would not be practical due to the greater length, but viral expression approaches used for miRNAs could well be adapted for lncRNA delivery. However, as some models propose lncRNAs function through recruiting proteins to the nascent transcript, the site of expression may be critical for their functions. Ectopic expression may not be able to recapitulate the full effects of endogenous lncRNAs. Another concern is the stage of development at which lncRNAs act. T2D susceptibility at the *KCNQ1OT1* locus involves the imprinting, which is lost in adult beta cells, suggesting that this lncRNA plays a role in beta cell development. It is therefore possible that manipulation of *KCNQ1OT1* in adult beta cells would be unable to ameliorate any developmental defects. To counter this view, there are a large number of lncRNAs expressed in adult beta cells, including some only found in mature beta cells. Furthermore, the regulation of *GLIS3* by one islet lncRNA demonstrates that significant diabetes genes are regulated in adult beta cells by lncRNAs (Morán et al., 2012).

## CONCLUSION

The large number of highly cell-type specific lncRNAs identified in recent years provides the potential for significant regulation of gene expression and cell phenotype in pancreatic beta cells. However, the functional characterization of these lncRNAs has inevitably lagged behind their discovery. So far there is evidence for the epigenetic regulation of two highly significant T2D loci by lncRNAs. In addition there is more circumstantial evidence that a number of further lncRNAs are associated with beta cell development and function. Whether lncRNA expression changes during beta cell compensation to insulin resistance, potentially playing a role in beta cell expansion under these conditions, is also an important area for investigation, and may reveal the potential for therapeutic targeting. Further detailed investigation of the specific lncRNAs involved in this process are required to reveal the true extent to which lncRNAs regulate beta cell identity, proliferation and function.

## Conflict of Interest Statement

The authors declare that the research was conducted in the absence of any commercial or financial relationships that could be construed as a potential conflict of interest.
